# How does the developing mind construct a self?

**DOI:** 10.1098/rstb.2025.0158

**Published:** 2026-11-10

**Authors:** Victoria Southgate

**Affiliations:** Department of Psychology, University of Copenhagen, Copenhagen, Denmark

**Keywords:** infants, development, self-awareness, self-concept

## Abstract

Our capacity to think about ourselves is a developmental achievement that, when it emerges, profoundly changes cognition. Currently, there are few, if any mechanistic accounts of where a concept of self could come from, and empirical investigation within contemporary developmental science is surprisingly absent. Drawing on a wealth of data from cognitive neuroscience and developmental psychology, I propose a new mechanistic account of the ontogeny of our reflective self-awareness anchored in infants' interoceptive signals. This is a social cognitive account in which infants' expectations about others' attention are deeply entwined with the modulating effect that others' attention has on infants' awareness of their interoceptive state, together providing the impetus for infants to generate a conceptual representation of self de novo.

This article is part of the discussion meeting issue ‘From being to identity: analysis and synthesis of the self’.

## 1. Introduction

Our capacity for self-awareness is often considered a defining feature of human consciousness [1,2]. While many species are probably conscious [3], the ability to subject oneself to conscious awareness and to think *about* oneself is often described as a uniquely human feat [1,4]. In adulthood, this capacity for representing the self as an object of thought probably allows us to, among other things, imagine how others see us, scrutinize our feelings, recollect our past and contemplate our future. This kind of self-related process entails reflecting upon, representing or thinking about the self, and arguably relies upon a conceptual representation of self. Although a self-awareness of this sort may be the *sine qua non* of human experience and critical for our success as a species [5,6], this distinctive feature of human consciousness is probably absent at birth. Despite some highly important work characterizing the nature of self-awareness in infancy [7,8], there are few, if any, mechanistic theories identifying candidate cognitive and neural mechanisms that may contribute to its emergence and development.

Discussions of the self in philosophy and cognitive science often invoke different kinds of selves [9] or a layered or hierarchical structure of increasingly abstract selves [10–12]. While such taxonomies have provided valuable insights into different facets of self-related processing, and some distinctions may be supported by neuroimaging data [13], they are typically framed with reference to mature cognition (see Rochat [11] for an exception). As such, they do not necessarily map onto the self in *development*. For example, empirical work suggests that adults can have fundamental impairments in episodic memory but intact self-representation. This allows the identification of preferences or feelings in the absence of any episodic information, suggesting that our mature self-representation does not require reference to specific episodes [14]. However, this tells us little about how episodic memory might contribute to the development of our self-representation. Indeed, there is evidence for a close relationship between episodic memory and self-knowledge in early development [15,16], and it is plausible that episodic memory is foundational for the development of a self-concept, providing necessary input to link the self at different points in the past to construct a concept of self. However, as the self-concept matures, episodic information that was crucial to its construction may no longer be required for its function. Thus, we should be cautious in applying to development distinctions regarding the self in adulthood.

Nevertheless, some distinctions seem ontogenetically probable. Both Neisser [17] and Rochat [11] referred to a stage in which a *concept* of self emerges. A concept is an amodal representation that refers to a kind that can be evoked across situations even when the relevant stimulus or feeling is absent, and which supports categorization, generalization and inference. A concept is, by definition, an abstraction. Concepts are decouplable from the here-and-now and need not be tied to any particular sensation or modality [18,19]. Thus, a concept of self would allow an infant to think about itself. From this definition, it should be clear that numerous self-specifying signals (e.g. multisensory redundancy, efference copy, proprioceptive feedback) exist and effectively guide action prior to the availability of any conceptual representation, which may include the ability to redescribe those signals as belonging to the self [20]. Once available, conceptual content develops through increasing elaboration and abstraction, and so definitions of what constitutes a self-concept must also be broad enough to cover the birth and continued abstraction of the concept. A concept of self for Neisser evokes an object of reflection that includes one's traits, identities and roles [17], whereas Rochat and others referred to conceptual aspects like continuity and distinctiveness, which probably developmentally precede any inclusion of identity or roles, but nonetheless still constitute conceptual information [7]. From a developmental perspective, a concept emerges as an organizing structure that becomes more elaborate over time, as it is enriched with additional exemplars, distinctions and relations. Thus, to talk of a self-concept at 18 months need not imply that initially this concept contains details of one's perceived identities or preferences, but only that such a concept now exists and has the *potential* to contain such information. Initially, a concept of self may be only a representation of self as an object that can perceive something and be perceived by others, right now. The same concept may later support a representation of the self as an entity that perceived something in the past and could perceive things in the future or was perceived by someone else in the past. Conceptual development unfolds when a concept is modified or updated, depending on experience and new input [18]. Thus, it is unclear—from a developmental perspective—that different discrete levels of self should be extracted from the existence of a conceptual representation that is simply elaborated over time. While increasing elaboration and abstraction of the concept may permit the child to engage in different behaviours, some of which may represent genuine qualitative changes in self-awareness (such as a representation of one's perception in the here and now versus a representation of one's perception across time), the conceptual representational nature of self exists already. Thus, an emerging conceptual representation may, at first, be a skeleton, but this concept will eventually contain all the information about our identities, preferences and beliefs. It is the developmental origin of this skeleton self-concept—the point when the child has a concept when before it did not, and as a result can now *think about itself*—that I wish to account for with the model that I will present in this paper. It is this concept of self that may well be absent in most other species and that may mark the difference between consciousness in humans and other animals.

## 2. The initial absence of self-representation

The historical consensus that we operate with at least two broad kinds of self-awareness [9,10,21] has contributed to research in developmental science that has assumed a *bodily* self-awareness that precedes a *mental* self-awareness. Scholars like Rochat have provided a rich descriptive map of the developmental trajectory of self-awareness, hypothesizing an innate foundational core giving implicit self-awareness of the body that, by around 18 months, has grown into a conceptual representation of the self [11,22]. This conclusion is partly based on infants’ success at around this age on the mirror self-recognition (MSR) test, in which a mark is covertly placed on the infant's face and self-representation attributed if, when placed in front of a mirror, the infant attempts to touch the mark [23]. In Western cultures, roughly 50% of infants do this by 18 months, and by 24 months almost all infants will do this [11,22,24]. Although the relevance of MSR for conceptual self-knowledge has been challenged [25,26], there is an extensive research literature linking MSR to a variety of behaviours that ostensibly imply a capacity for self-representation [27]. For example, children who pass the MSR test are more likely to use personal pronouns [28], to show self-conscious emotions like embarrassment [29] and to exhibit empathy [30]. In our own work, we have shown that the absence or presence of MSR systematically predicts infants’ encoding of other-related or self-related events, respectively. At 18 months, infants who do not yet recognize themselves in a mirror prioritize encoding of objects assigned to others, a possible manifestation of the altercentric bias we had previously documented in younger infants [31]. However, 18-month-olds who already recognize themselves in the mirror showed a prioritized encoding of objects assigned to themselves [32], similar to the self-reference effect characteristic of adult memory [33]. In another paradigm, 18-month-old mirror self-recognizers exhibited evidence of experiencing a conflict when self and other perspective diverged, whereas 18-month-old non-recognizers showed no evidence of experiencing such a conflict [34,35]. Neither of these effects was explained by general cognitive development, as in other measures there were no differences in infants who did or did not recognize themselves in the mirror. Finally, we also found that 18-month-old mirror self-recognizers had significantly greater fronto-temporoparietal resting state functional connectivity than age equivalent non-recognizers [36]. This activity pattern overlaps with regions of the cortex that together compose the default network, implicated in self-referential processing in adults. Thus, there is now ample evidence that something profound changes in the second year of life in infants’ capacity for thinking about themselves [37].

Although Rochat conceived this development of self-awareness as an additive layering of increasingly abstract representations upon a biologically innate core [38], no specific mechanisms are hypothesized that might drive this layering process. Nonetheless, many scholars have proposed that a self-awareness is interpersonally constituted through the typical interactions between infants and their caregivers [39–42]. For example, both Atzil *et al.* [39] and Fotopoulou & Tsakiris [40] target caregiving inputs (e.g. touch, holding, feeding and synchrony), and argue that their modulation of infants’ interoceptive states (arousal, hunger, pain, temperature) provides the data that calibrate interoceptive predictions and, in doing so, could provide a pre-reflective sense of ‘mineness’ [40]. Gergely & Watson [41] propose that infants become aware of their emotions through detecting the contingency between marked mirroring by the caregiver of the infant's internal states. According to this model, over repetitions, infants build secondary representations of each emotion that can be reactivated without the caregiver, enabling self-attribution and control of emotion. Most relevant for the current proposal, Reddy also proposes that a preconceptual self emerges through social interactions and specifically through infants as the object of the caregiver's attention [42]. Each model offers valuable frameworks for thinking about how pre-conceptual aspects of self (e.g. interoceptive prediction and affect regulation), rather than being present at birth, could depend on different components of caregiver–infant interactions. However, none of these models offers a route to a conceptual self-representation that could allow infants, eventually, to self-ascribe these self-specifying signals. Given that it is this conceptual representation of self that may differentiates human self-awareness from that of other species, I believe the challenge of developmental science is to show how non-conceptual self-related information could plausibly become a conceptual self-representation [20].

In the current paper, I propose a *mechanistic* account of how infants’ early experience of being the target of others’ attention could provide the necessary conditions for not only a pre-conceptual self-specifying *feeling* [42], but also a pathway beyond this to a conceptual representation of the self. The account takes the social modulation of internal states as the foundation and proposes a pathway through which infants become aware of their interoceptive states through their modulation by caregiver ostensive cues. Crucially, I propose that the *non-conceptual to conceptual transition* occurs as a result of the expectations that the developing human mind has about the referential nature of ostensive cues, providing a plausible route to a concept of self.

## 3. Interoceptive origins of self-awareness

Interoceptive signals are those that arise from within the body, like heartbeat. There is now substantial evidence that sensitivity to, or awareness of, heartbeat is linked—in some way—to our self-awareness. For example, performance accuracy on a heartbeat discrimination task positively correlates with the intentional binding effect [43], suggesting that there may be a relationship between interoceptive sensitivity and our sense of agency. Performance accuracy on heartbeat discrimination also correlates with the amplitude of ERPs (event-related potentials) implicated in behavioural monitoring and metacognition [44]. Both of these effects are typically interpreted as indexing some element of self-related processing, and these associations with heartbeat awareness suggest a link between interoceptive sensitivity and core self-related functions. There is also evidence showing that an individual's interoceptive accuracy predicts the malleability of the self-representation in the rubber hand [45] and enfacement illusions [46]: those with weaker interoceptive awareness are more susceptible to self–other overlap. Furthermore, in an enfacement illusion paradigm, synchronizing a face's visual pulse with the participant's heartbeat shifted self-face recognition towards judging morphed faces as ‘self’; indicating that aligning external cues with cardiac signals can bias the self–other boundary towards the self [47].

The heartbeat is also represented cortically in the heartbeat-evoked response (HER), including in the insula [48], and can be measured as the heartbeat-evoked potential (HEP) with electroencephalography (EEG). The HER is also modulated by self-relevance and its amplitude is enhanced in response to numerous self-related events, including when we engage in self-related thoughts [49], hear our name [50] or prepare to make a subjective choice [51]. There is also work showing that presenting a self-relevant stimulus at systole (rather than diastole) facilitates self-referential processing [52]. As systole activates the baroreceptors that may be one route to the generation of the HER [53], this could suggest that the HER is involved in interpreting stimuli as self-related.

Several scholars have suggested that our visceral signals could provide the foundations of a conceptual self-awareness [54], but how this could happen remains unclear. An intriguing account hypothesizes that self-specificity may be implicitly encoded within the HER. As the HER is modulated by self-relevance, it has been hypothesized as a plausible mechanism to bridge, perhaps by virtue of insula connectivity [55], from an implicit self-specifying signal to an awareness of those signals [56]. Damasio's somatic marker hypothesis proposes ‘feeling states’, or the subjective, conscious experience of emotions, as an intermediary between interoceptive signals and a self-awareness, proposing that self-awareness unfolds when this feeling state can be represented as engaged and modified by an object [54]. This implies that a conscious self-awareness arises when we notice the feeling state and we can connect it to the cause that gave rise to the feeling state.

Infants too are sensitive to their interoceptive signals and discriminate between an animated shape that bounces asynchronously with the infant's heartbeat and one that bounces synchronously [57,58]. That infants detect congruence between their heartbeat and an external stimulus suggests that they have a representation of their heartbeat that is sufficiently abstract to detect a corresponding visual representation. In infants, the amplitude of the HEP was also higher in those infants who showed greater discrimination between the heartbeat-synchronous and -asynchronous bouncing events [58], suggesting a relationship between interoception and the HEP in infants similar to that in adults [59]. In the next sections, I will put forward an account of a possible pathway through which the infant's heartbeat could serve as the input to a pathway that will ultimately lead to a concept of self, via the modulating effect of caregiver communication, and expectations that young infants already have about the referential nature of others communication.

## 4. The modulating effect of others' attention

An intriguing aspect of interoceptive awareness is that it seems to be enhanced by the presence of others’ attention to oneself. For example, participants are more accurate on the heartbeat-counting task when they are presented with a face with direct rather than averted gaze [60], and participants with higher heart rate variability are more likely to report themselves as the target of others’ gaze when that gaze is presented at systole, when cardiac afferent signalling to the brain is increased [61]. This effect may reflect the broader phenomenon of eye contact inducing self-awareness [62]. Several studies suggest that the presence of eye contact elicits greater bodily self-awareness by enhancing awareness of one's physiological states [63] or blurring the boundary between self and other by lowering the point of subjective equality when perceiving another face making eye contact [64]. These effects are consistent with the relationship described above, between the HER and more abstract aspects of self-awareness. If direct gaze is perceived as self-relevant and the heartbeat-evoked response is modulated by self-relevance, or the heartbeat-evoked response fosters a feeling of self-relevance, then the effect of direct gaze on bodily self-awareness, including interoceptive awareness, makes sense.

But the very interpretation of direct gaze as self-relevant presupposes a sense of self. If human infants initially do not have self-awareness, how could they acquire the understanding that others’ eye contact is self-relevant? There is a long history of research addressing how human infants understand others’ gaze. Newborn infants detect direct gaze from birth, looking longer towards a face gazing directly at them compared with a face averting its gaze [65]. A face with direct gaze also evokes a larger-amplitude face-sensitive ERP, suggesting that direct gaze may facilitate face detection. It seems probable that early face detection is achieved via subcortical mechanisms that are tuned to face-like stimuli, and which, when accompanied by direct gaze, elicit further processing in the amygdala and the limbic system, as well as cortical processing by the superior temporal sulcus [66]. However, despite this early sensitivity to direct gaze, there is little evidence that infants interpret others’ gaze as self-relevant, and it is probable that this understanding needs to be acquired. I will propose that the answer to how this understanding could be acquired is also the answer to the question of how a self-awareness itself is acquired.

## 5. A proposed mechanistic account

As mentioned above, the presence of direct gaze activates subcortical structures like the superior colliculus via direct pathways from the retina. This subcortical pathway also involves the amygdala and hypothalamus, which activates the sympathetic and parasympathetic nervous system [67]. Studies show that direct gaze leads to changes in heart rate, both acceleration and deceleration [68], reflecting sympathetic and parasympathetic influences on cardiac activity. In infants, heart rate acceleration in response to direct gaze, but not averted gaze, has been interpreted as reflecting autonomic arousal, preparing the infant for social engagement and learning [69].

 Inspired by Damasio's [54] proposal that self-awareness results from changes in interoceptive states by way of the representation of feeling states changed by an external object, I propose that direct gaze is just such an external object that could function to alter the infant's interoceptive states and bring them to awareness. According to predictive coding accounts, because the strength, rhythm and variability of our heartbeat is constantly present, it can be fully predicted and so will usually result in no awareness. However, when contexts induce unpredicted changes in heart rate, these signals are more likely to reach awareness [70]. Heartbeat may be the most important signal for initiating the self because it is present before the brain develops and provides a constant signal [71].

Initially, there may be barriers to infants’ ability to exploit their interoceptive signals, including heartbeat. Infants develop within another body, which also has a heartbeat, and then spend considerable time—depending in part on culture—in close contact with another body. As several pathways can provide heartbeat information, including projections to sensorimotor cortex from skin vibrations [72,73], early attempts to internally model biological signals, like heartbeat, may incorporate salient signals from the mother or caregiver [74]. However, once infants gain more bodily distance from the caregiver and cleaner interoceptive data are detectable, caregiver social communicative behaviours, like direct gaze, which lead to changes in heartbeat, could bring these signals to infants’ awareness. While the current proposal is inspired by extensive empirical work on direct gaze, other social communicative behaviours, including infant-directed speech and affective touch—all part of the human caregiving repertoire—also lead to changes in heart rate in the infant recipient [75–77]. Presumably, at some point, the infant brain will learn to expect these changes and incorporate them so that they are fully predicted (i.e. result in no awareness), but initially these changes could be the data that infants can exploit to build an interoceptive awareness. Interestingly, in one of the few demonstrations of interoceptive awareness in infants to date, interoceptive accuracy was associated with the extent to which six-month-old infants experienced episodes of direct gaze with the caregiver [69]. This would be consistent with the suggestion here, that sensitivity to interoceptive signals develops as a consequence of caregiver communicative signals that trigger changes in interoceptive data. This pathway would provide a means by which infants are alerted to their heartbeat. If indeed the HER is an inherently self-specifying signal [13], then it could tag these detectable changes as *belonging to the body.*

A sense of *belonging to the body* is central to Damasio's notion of *feeling states*. Feelings are states of which we can be aware, which are generated by interoceptive states of which we are not aware, for the function of homeostatic–allostatic regulation [1,78,79]. For Damasio, functional self-specification is part of such feeling states because they are maps of the state of one's own body and thus their content is intrinsically about the organism experiencing them. Various models propose that feeling states are derived from second-order representations of primary interoceptive activity [54,78], and that the anterior insula, owing to its unique hierarchical architecture as well as connectivity with prefrontal cortex, makes a good candidate for this process [55], which may be uniquely human [78].

As already discussed, Damasio has proposed that self-*awareness* unfolds when this *belonging to the body* feeling state can be represented as engaged and modified by an object [54]. Applying this idea to development, I propose that, when direct gaze induces a change in the infant's heartbeat, the accompanying HER enhances the specificity of that feeling, marking it as self-related and embodied. If infants notice that this feeling state proceeds from direct gaze, direct gaze will initially be bound up in the infant's developing internal model of their heart rate. This association (direct gaze–heartbeat-feeling state) links the perception of direct gaze to the implicitly self-specified feeling (*belonging to the body*) state encoded in the HER. Assuming that, with sufficient experience, infants can predict the heart rate change accompanying direct gaze, it will no longer lead to awareness. But, by this point, the association between direct gaze and the *belonging to the body* feeling state engendered by the HER provides a direct link. This pathway provides a route through which infants become aware of their heartbeat, and it gives rise to a *belonging to the body* feeling state.

Nevertheless, this embodied feeling does not constitute a *concept* of self. From this pathway, while infants would have made an important prerequisite link between direct gaze and an inherently self-specific feeling, this is not a conceptual understanding, or one that allows infants to understand that others’ eye contact is self-referential. It probably comes closer to a kind of minimal, pre-reflective self or proto-self, which scholars like Zahavi describe, though under the current account some of this process would be a developmental achievement, rather than an innate prior [80]. However, the current model proposes a way in which this embodied self *feeling* can transform into a conceptual self through the expectations that infants have about others’ gaze (figure 1).

**Figure 1.**
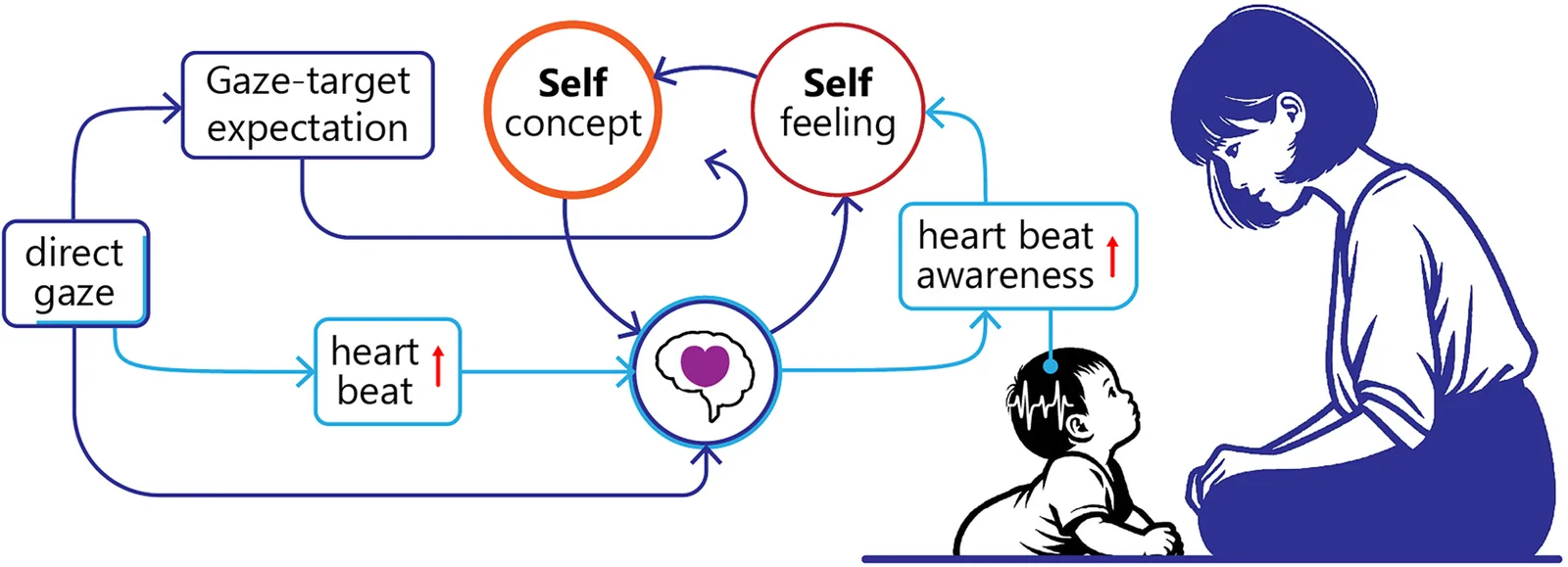
This figure illustrates the pathway through which eye contact could give rise to a self feeling state via its modulating effect on the heartbeat and the heartbeat-evoked response (light blue lines). If infants can detect that heartbeat increase is caused by eye contact, this learning would correspond to a process that imbues direct gaze with the same feeling (dark blue lines). This, together with a bias to seek the targets of others’ gaze, may allow infants to link an initial placeholder referent with the self-feeling, giving rise to the origins of the self-concept. The heartbeat-evoked response (HER), self-feeling, self-concept loop captures the finding in adults that the HER can be amplified by evoking the self-concept and suggests that developmentally this could give rise to an accumulation of evidence for a distinct category for the self.

## 6. Eye contact as *about me*

A long-standing debate within the developmental field concerns when, during ontogeny, infants’ gaze-following reflects an understanding that gaze is referential [81]. By eight months, infants show some understanding that gaze is referential, under some conditions [82,83] and a robust understanding of gaze as referential is evident by the end of the first year of life [84].

Once it is available, the expectation that others' gaze is related to targets and the motivation to identify the targets of others' gaze could provide a way in which infants come to identify themselves as the target of others' eye contact. Initially, infants would not know that they are the target of eye contact, but their motivation to identify the target of others' gaze will lead them to seek such a referent and, possibly, generate a placeholder referent [81]. In the same way as they expect ‘something’ when an agent looks behind an occluder [82], they should expect ‘something’ when the agent looks at the infant. This placeholder is not just a gap in perception but a conceptual space: an abstract representation that marks ‘there is something to be found’. As this space is inherently conceptual, infants will seek to fill it with a concept.

The infant now has two simultaneous responses to being the target of others' gaze. First, it experiences a functionally specified belonging to the body feeling state owing to a prior association between eye contact and changes in the heart rate. Second, it has an expectation that the gaze must have a target. The claim I make here is that, once an association is generated between direct gaze and the *belonging to the body* feeling state, infants can fill this conceptual placeholder with this feeling state, which provides the origin of a conceptual representation of self.

Several factors probably influence the timeline of this trajectory. Clearly, the experience of things like eye contact is central to this account, and so marked differences in the degree of ostension between infants and their caregivers would be predicted to alter the timeline. In support, there are differences in opportunities for eye contact across cultures that may map onto differences in the acquisition of a conceptual representation of self. For example, infants in several non-Western cultures are reported to pass the mirror self-recognition test later than their counterparts in Western cultures, and for several of these populations there are separate documented differences in their experience of eye contact (e.g. Vanuatu [85]; Congo [86]). In one study, mutual eye contact at three months of age actually predicted mirror self-recognition at 18–20 months [86]. While eye contact is by no means likely to be the only relevant cue that can function in the proposed model, I argue that it is probably the most powerful of communicative cues because it has a clear biological route through which it can affect the heartbeat, and infants have early expectations about its referentiality. Furthermore, it is a near-universal [87] in human caregiving, and its absence is very rare. As acknowledged, other infant-directed behaviours like infant-directed speech and affective touch have similar modulating effects on the infant's heartbeat. Infant-directed speech seems to function similarly to eye contact in generating an expectation of content [83]. While I am not aware of any data on infants’ interpretation of affective touch as referential, others have speculated that touch is processed as a social signal by infants. For example, four-month-olds do not encode facial identity if it is not preceded by direct gaze [88], but if gaze is substituted for affective touch, infants encode facial identity [89].

Another factor that probably influences this timeline is brain maturation and developing structural and functional connectivity between the insula and prefrontal cortex (PFC) [90]. Others have made the claim that structural connectivity is necessary for a feeling state to be second-order represented [55], and that this connectivity pattern may be unique in humans. Anterior insula cortex is core to the processing of interoceptive as well as other self-related signals, and recent models identify the insula as commonly activated across multiple, increasingly abstract levels of self-processing [91]. As self-processing moves beyond interoceptive signals and becomes increasingly abstract, regions including temporoparietal junction (TPJ) and PFC also become involved, with insula–PFC anatomical connectivity proposed to allow higher-order interoceptive representations [55]. Gaze shifts towards a participant increase functional connectivity between other social brain regions and the anterior portion of the insula, and the social aspect of direct gaze is hypothesized to be processed by direct fibre connections between right posterior superior temporal sulcus (STS) and right anterior insula [92]. Functional magnetic resonance imaging (MRI) during the first 2 years of life reveals dramatic development in connectivity patterns associated with different insula regions [90], and functional connectivity involving the insula is modulated by caregiver behaviour [93]. We should expect that infants’ developing awareness of their interoceptive signals will depend on sufficient structural and functional connectivity within and between the anterior insula and other cortical regions, as well as the microstructure of the default network.

## 7. Conclusions

Our capacity to think about ourselves, while clearly central to the human experience, is probably a developmental achievement. Yet, although it is a fundamental biological principle that, to understand any behaviour, we need to ask how it develops [94], the question of how a conceptual self-representation emerges during ontogeny has been neglected in developmental science, with a few notable exceptions. The proposal put forward in this paper is an initial attempt to formulate a biologically plausible mechanism that is open to empirical testing. This proposal shares with other frameworks an assumption that early caregiving interactions create the context for the emergence of self-awareness, and do this by affecting infants’ internal states [39–42]. However, while other accounts have sought to explain non-conceptual aspects of self-awareness—what the field often describes as a minimal, affective self—the current account aims to explain how these same visceral signals, in the context of uniquely human communication, could also translate non-conceptual to conceptual self-representation. Along with others (e.g. [20]), I believe this is the biggest open question for our understanding of what is unique in our human capacity for self-awareness. The current model leans on the functional self-specificity of feeling states [54] and proposes that, because one and the same signal (gaze) brings both non-conceptual content (feeling states) and conceptual expectation (referentiality), it could function as a bridge between nonconceptual and conceptual representation, leading to the possibility that non-conceptual content gets redescribed as conceptual content. Thus, in the search for conceptual content, the simultaneously available non-conceptual content most probably populates this empty placeholder.

To be useful, this account must explain both cross-cultural variation within our own species [95] and the probable absence of a conceptual self-representation in many other species [96]. The priority given to direct gaze in the current model can potentially accommodate both phenomena. First, parenting in which face-to-face interaction *dominates* is not a cultural universal [97,98]. Second, while mutual gaze does occur within mother–infant dyads in some non-human primate species, such events are fewer and of shorter duration [99]. An interesting exception that is relevant here may be dogs, for which mutual gaze may, as in human infants, be understood as referential [100–102], and for which there is some intriguing evidence of an understanding of different kinds of selves [103,104].

Finally, the central role of ostension, and in particular direct gaze, in this model need not imply that the constitutive components of the model evolved to support a reflective, conceptual self-awareness in humans. Eye contact has long-standing biological functions, including rapidly modulating the receiver's arousal and autonomic state via subcortical pathways associated with social relevance and vigilance, probably selected for threat detection, affiliation and attention capture [105]. In humans, the same cue is additionally exploited as an ostensive, referential signal that marks a specific addressee and highlights what is to be learned, supporting efficient cultural transmission [106]. It may simply be that these two components interact during early development in a way that creates conceptual self-awareness as an emergent by-product, rather than as a specifically selected function of either system.

Notwithstanding the enormity of the challenge to elucidate the origins of a conceptual self-awareness in human development, and to understand why this capacity emerges in only very few species (if any, beyond humans), the current model is proposed—in part—to foster a renewed interest in the topic of self-representation in early development, and to move the field towards mechanistic accounts and testable hypotheses.

## Data Availability

This article has no additional data.

## Theme

One contribution of 17 to a discussion meeting issue ‘From being to identity: analysis and synthesis of the self’.
